# Advances of paeoniflorin in depression: the molecular mechanism and formula application

**DOI:** 10.3389/fphar.2025.1614429

**Published:** 2025-09-03

**Authors:** Yi Hou, Hong Li, Luochen Zhu, Tian Quan, Xianhu Feng, Yue Li, Yuan Bian, Yuxun Wei

**Affiliations:** ^1^ Pharmacy Department, The Center of Clinical Trials, The People’s Hospital of Zhongjiang, Deyang, China; ^2^ West China School of Medicine, Sichuan University, Chengdu, China; ^3^ Department of Pharmacy, Nanjing University of Chinese Medicine, Nanjing, China; ^4^ Department of Pharmacy, Nanchong Central Hospital, North Sichuan Medical College, Nanchong, Sichuan, China; ^5^ Molecular Urooncology Department of Urology Klinikum rechts der Isar Technical University of Munich, München, Germany; ^6^ Department of Oncology, Xichang People’s Hospital, Xichang, China

**Keywords:** paeoniflorin, medicine formula, depression, pharmacological mechanism, pharmacokinetics

## Abstract

Paeoniflorin (PF) is widely present in species of the *Paeonia* genus. In recent years, numerous preclinical studies have shown that PF has preventive and therapeutic effects on various neurological diseases, particularly in the prevention and treatment of depression. Additionally, some classic traditional Chinese medicine formulas containing PF, such as Xiaoyao San, Chaihu Shugan San, and Sini San, have been proven to significantly improve depressive symptoms. However, the antidepressant mechanisms of PF and its containing classic traditional Chinese medicine formulas are not yet fully understood. PF, as a natural glycoside metabolite with a wide margin of safety and good tolerance, exhibits certain toxicity at high concentrations. The differences in standardized methods between the traditional formulations, such as extraction processes, dosages, and inherent metabolite variability in formulations, may affect the interpretation of results and clinical applications. Therefore, this article reviews the antidepressant mechanisms of PF from the perspectives of inhibiting the hypothalamic-pituitary-adrenal axis, increasing the levels of monoamine neurotransmitters, suppressing oxidative stress and apoptosis, regulating calcium homeostasis, inhibiting neuroinflammation, modulating mitochondrial function, regulating cellular autophagy, and increasing the levels of brain-derived neurotrophic factor, and elucidates the antidepressant effects and mechanisms of traditional Chinese medicine formulations containing PF. Additionally, we describe the physicochemical properties, toxinology, pharmacokinetic characteristics, and the transformation of PF *in vivo*. This review may contribute to the application of PF and its formulations in depression.

## 1 Introduction

Depression, the most prevalent mental disorder, imposes a huge burden on individual health and wellbeing as well as social and economic development, which has become one of the major global mental health challenges. According to Global Burden of Diseases (GBD) data survey statistics, depression is one of the leading causes of disability-adjusted life years (DALYs) for people aged 10–49 years (10–24 years: fourth, 25–49 years: Sixth) (Diseases and Injuries, 2020). When only mental disorders were considered, depression ranked highest of DALYs in all age groups except those aged 0–14 years, as behavioral disorders were the main cause of burden (Collaborators, 2022; Whiteford et al., 2013). According to the World Health Organization (WHO), depression will become the leading cause of death worldwide by 2030 (Tian et al., 2013). Depression, as defined by the American Psychiatric Association in 2013, is a common and profoundly heterogeneous disorder (Johnston et al., 2019), usually accompanied by physical, behavioral, and psychological symptoms such as changes in appetite, headaches, sleep disturbances, persistent low mood, lack of pleasure, sexual problems, and suicidal ideation (Marwaha et al., 2023; Disner et al., 2011). These physical and emotional disorders seriously affect the work and daily life of individuals, and significantly reduce their quality of life and happiness. Therefore, the search for effective treatment should be an important research topic.

Brain stimulation, including repetitive transcranial magnetic stimulation, transcranial direct current stimulation, and deep brain stimulation are common treatment for depression nowadays (Marwaha et al., 2023). However, regrettably, these seemingly attractive treatment methods can still trigger a series of side effects, including headaches, scalp discomfort, fatigue, pain, dizziness, insomnia, eye and nose issues, as well as gastrointestinal problems (Miuli et al., 2021; Hett and Marwaha, 2020; Chen L. et al., 2020; Moffa et al., 2020; Berlow et al., 2019; Zhou et al., 2018; Dougherty et al., 2015). Due to the time and economic loss caused by the treatment, the benefit to the audience is limited. Medication still seems to play an irreplaceable and important role in severe cases of depression. In clinical settings, antidepressants such as TCAs, MAOIs, selective 5-HT reuptake inhibitors, and 5-HT-NE reuptake inhibitors are widely used for the treatment of depression. However, these traditional antidepressants still have inevitable side effects and drug-drug interactions, such as dry mouth, blurred vision, inability to drive, sexual dysfunction, decreased libido, headache, gastrointestinal symptoms, anxiety, agitation, and other common adverse events (Ray et al., 1987; Roose et al., 1994). More disappointingly, less than half of the patients treated with these medications show a beneficial therapeutic response and induce drug tolerance if used for a prolonged period of time (Cipriani et al., 2018; Rush et al., 2009). Therefore, there is an urgent need to develop antidepressant medications that act more rapidly, have better tolerability, offer superior therapeutic efficacy, and are associated with fewer side effects.

Numerous studies have shown that Complementary and Alternative Medicine (CAM) has extensive application value in the field of mental health treatment, particularly in the treatment of depression (Kessler et al., 2002; Unutzer et al., 2000; Knaudt et al., 1999). Natural drugs as alternative treatment methods may be a promising attempt, which have significant therapeutic effects, relatively small side effects, and low prices. Certain extracts or single units of natural medicines have shown great potential to treat psychiatric disorders, such as PF (Peng et al., 2022; Hong et al., 2022; Guo et al., 2022).

**FIGURE 1:**
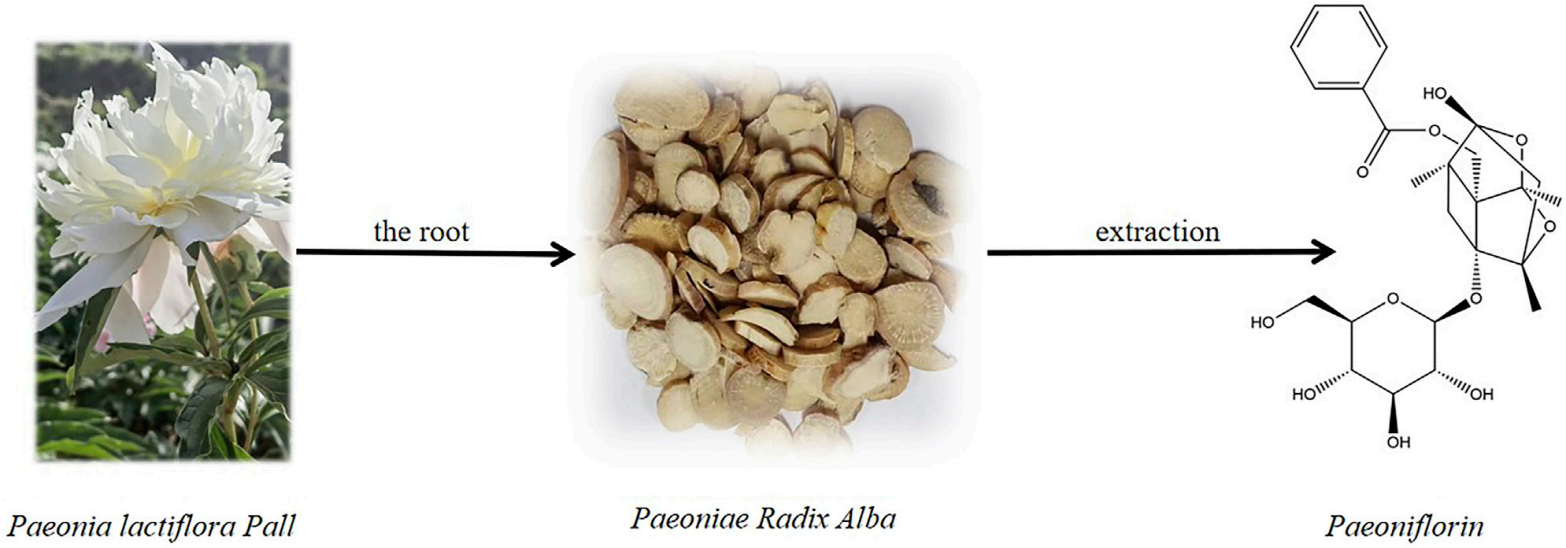
Paeoniflorin and the source from the roots of *Paeonia lactiflora Pall*.

Paeoniflorin (PF; C_23_H_28_O_11_) is a water-soluble monoterpene bicyclic glycoside extracted from *Paeonia lactiflora* Pall. [Paeoniaceae, *Paeonia lactiflora root*] is a plant known for its medicinal and edible properties. First isolated in 1963, its chemical structure was determined in 1972 (Wang Z. et al., 2022) (Figure 1). As an additional nutritional metabolite in plant-based foods, PF offers health benefits that extend beyond basic nutritional value, with increasing evidence supporting its positive impact on human health. In recent years, most studies have found that PF has a wide range of pharmacological effects *in vitro* and *in vivo*, including anti-inflammatory (Tu et al., 2019; Xin et al., 2019; Wang C. et al., 2013), anti-oxidation (Chen et al., 2011), anti-thrombosis (Ye et al., 2016), anti-convulsion (Hino et al., 2012), analgesia (Zhang et al., 2009), cardioprotection (Chen H. et al., 2018; Wang et al., 2020), neuroprotection (Mao et al., 2012a; Kong et al., 2020), liver protection (Jiang et al., 2014), antidepressant (Qiu et al., 2013; Cheng et al., 2021), anti-tumor (Lu et al., 2014), immunomodulation and so on (Wang Z. et al., 2022; Zhang and Wei, 2020). PF can be used as a potential therapeutic agent for many diseases, such as psoriasis (Bai et al., 2020), atherosclerosis (Yu et al., 2022; Duan et al., 2021), and depression (Hong et al., 2022; Guo et al., 2022), due to the inherent advantages of low toxicity, high efficiency, and safety. The intrinsic pharmacological mechanism of PF has received great attention from researchers and clinicians. Currently, more and more evidence suggests that PF has significant pharmacological activity against various neurological diseases, for instance, cerebral ischemia (Wu et al., 2020; Guo et al., 2012; Liu et al., 2005), vascular dementia (Luo et al., 2018; Zhang et al., 2016), Parkinson’s disease (Zheng et al., 2017; Liu H. Q. et al., 2006), and depression (Li et al., 2020; Chen et al., 2019). Especially for depression, PF may exert surprising antidepressant effects through oxidative stress, apoptosis, neuroinflammation, and other mechanisms (Guo et al., 2022). In traditional Chinese medicine, some prescriptions containing PF, such as Xiaoyaosan (XYS) (Chen C. et al., 2020), Chaihu-Shugan-San (CSS) (Kim et al., 2005), and Sinisan (SNS), have been proven to have significant improvement effects on depression and are used as an alternative treatment for depression. This study reviews the pharmacokinetic properties of PF *in vivo* and pharmacological mechanisms in the treatment of depression, further determines the clinical application of PF in depression, and provides literature support for its drug formation research.

## 2 Methods

A comprehensive search was conducted across popular and widely used databases, including PubMed, Web of Science, Scopus, Science Direct, Google Scholar, and CNKI, utilizing various search strings. The search terms included, but were not limited to, “paeoniflorin, physicochemical properties, pharmacokinetics, antidepressant mechanisms, hypothalamic-pituitary-adrenal (HPA) axis, monoamine neurotransmitters, oxidative stress, apoptosis, calcium homeostasis, neuroinflammation, mitochondrial function, autophagy, and brain-derived neurotrophic factor (BDNF),” as well as the application of paeoniflorin in traditional Chinese medicine formulations (e.g., XiaoyaoSan, ChaihuShuganSan, SiniSan, etc.). The search was conducted in both English and Chinese, relying solely on online databases without incorporating other physical sources. Inclusion criteria encompassed studies related to the aforementioned aspects of paeoniflorin, including basic pharmacological research, pharmaceutical research, clinical research, and other formally published literature. Exclusion criteria consisted of studies that were irrelevant or minimally relevant to the specified aspects of paeoniflorin, duplicate publications, low-quality literature, and studies for which full texts were unavailable.

## 3 Physicochemical properties and metabolism

**FIGURE 2:**
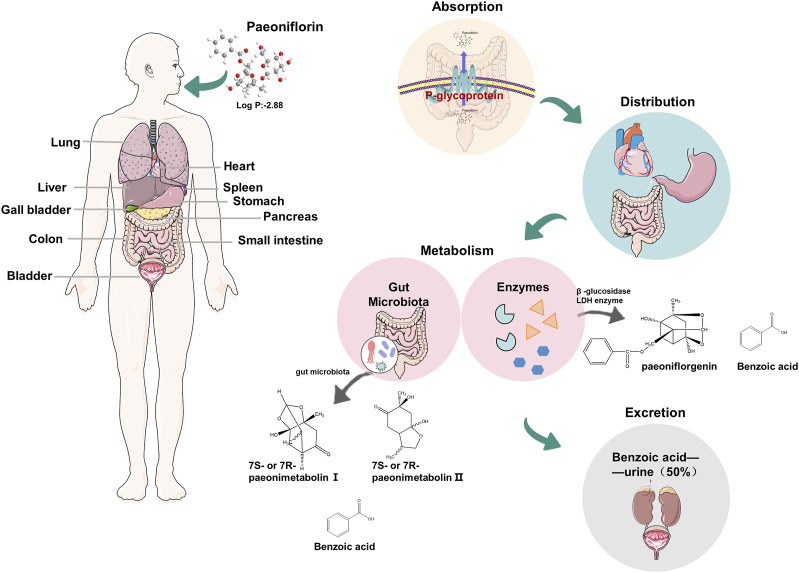
Physicochemical properties of paeoniflorin and the pharmacokinetics in humans.

Paeoniflorin (molecular weight 480.5) is a monoterpene glucoside, a strong hydrophilic plant metabolite (logP: 2.88). The pharmacokinetics study found that the low bioavailability of PF was about 3%–4%, related to the low permeability caused by the high hydrophilicity of PF. The absorption site of PF is mainly the intestine, and the absorption rate of the aglycone is 48 times that of PF (Takeda et al., 1995; Takeda et al., 1997; Yu et al., 2019) (Figure 2). Liu ZQ et al. demonstrated that the effect of P-GP efflux protein on PF and the metabolism of intestinal microorganisms is another important reason for the low bioavailability of PF (Liu Z. Q. et al., 2006). PF is widely distributed in various tissues after entering the systemic circulation, mainly in the stomach, intestine, and heart (Minmin Zhao et al., 2014). Significantly, PF has a smaller ability to penetrate the blood-brain barrier (permeability coefficient: 0.587 × 10^–6^ to 0.705 × 10^–6^ cm/s) (Hu et al., 2016). The metabolism of PF is divided into two pathways: intestinal flora metabolism and enzyme metabolism *in vivo*. After oral administration, the part of PF was metabolized into two chiral counterparts (7R or 7S paeonimetabolin Ⅰ, 7R or 7S paeonimetabolin Ⅱ) by the intestinal flora in the human being (Hu et al., 2016; Shu et al., 1987), and about 42% of the remaining PF was converted to paeoniflorgenin *in vivo* by β-glucosidase LDH enzyme (Hsiu et al., 2003). Interestingly, PF decomposes into paeoniflorgenin, similar to human fecal bacteria. Further studies have found that some bacteria can achieve mutual conversion of PF and aglycones. In addition, both pathways can metabolize PF to benzoic acid and can cross the blood-brain barrier to act on the central nervous system (CNS), which proves to some extent that PF has a certain neuroprotective effect (Yu et al., 2019). PF is mainly excreted in urine as benzoic acid after oral administration, and its cumulative excretion is 50%. On the contrary, less is excreted with the prototype drug, in urine, bile, and feces (Takeda et al., 1995; Yu et al., 2019), of which the property is inseparable from the low bioavailability of PF and the conversion of PF to benzoic acid by intestinal flora (Liu Z. Q. et al., 2006). Meanwhile, PF entering the systemic circulation is still mainly excreted in the form of urine.

## 4 Molecular mechanism of PF in depression

### 4.1 Hypothalamic-pituitary-adrenal axis

The hypothalamic-pituitary-adrenal (HPA) axis is one of the major biological stress response systems in humans, of which activation is essential to provide an appropriate biological response to stress (Guerry and Hastings, 2011). Activation of the HPA axis in stressful situations is a normal homeostatic mechanism that can contribute to the maintenance of stability and health by enabling adaptive changes in the body. For a healthy population, normal levels return when this stress response is no longer biologically significant. However, studies have found that abnormal activity of the HPA axis, such as excessive activity or persistent disorder, is closely related to the development of depression (Nelson and Davis, 1997; Curtis and Mendels, 1976; Bardeleben and Holsboer, 1989). Previous research has shown that the activity of the HPA axis is governed by vasopressin (AVP) and corticotropin-releasing factor (CRF) which is secreted by the hypothalamus and undergoes a cascade of events. Specifically, when the body is stimulated by stress, the adrenal glands secrete a surge of adrenaline, followed by the activation of the HPA axis. The hypothalamus secretes CRF for release into the portal circulation, and this in turn activates the pituitary to secrete the adrenocorticotropic hormone (ACTH), which ultimately stimulates the adrenal cortex to secrete glucocorticoids and cortisol (Pariante and Lightman, 2008). In addition, cortisol induces inhibitory feedback of the HPA axis by interacting with glucocorticoid (GR) and mineralocorticoid (MR), such as the secretion of ACTH, CRF, and AVP (Pariante and Lightman, 2008; Juruena et al., 2018; De Kloet et al., 1998). The normal physiological activity of the HPA described above maintains the relative homeostasis and health of the organism under certain stressful circumstances. However, excessive or prolonged psychological stress may alter the normal state of homeostasis in the body, which leads to depression, a serious mental illness that affects people’s mental and physical health. Studies have found that the most common biological abnormality in depression is HPA axis overactivity, which is characterized by increased cortisol, adrenal hyperplasia, and negative feedback abnormalities (Dinan and Scott, 2005; Dwyer et al., 2020). Fortunately, previous studies have shown that monoamine-based antidepressants can reverse stress-induced hyperactivity of the HPA axis (Seki et al., 2018). GR receptor antagonists, press-in receptor antagonists, and adrenocorticotropin-releasing hormone receptor antagonists can also exert antidepressant effects by blocking receptor activity to terminate hormone secretion resulting from HPA axis overactivity (Menke, 2019), suggesting that components of the HPA axis may be potential targets for the treatment of mood disorders (Thakore et al., 1997).

Most researchers suggest that PF has promising antidepressant activity by modulating HPA axis dysfunction. Qiu et al. found that intraperitoneal injection of PF (30 or 60 mg/kg) for 4 weeks significantly increased sucrose consumption, and decreased serum corticosterone (CORT) and ACTH levels in the chronic unpredictable stress (CUS) model group, which suggests that PF may exert antidepressant effects in CUS rats by regulating the HPA axis (Qiu et al., 2013). This preclinical study suggests and explores the antidepressant effects of PF and its underlying mechanisms; however, several significant limitations persist. Firstly, the generalizability of the findings is restricted due to the exclusive use of male SD rats as experimental subjects. Additionally, the CUS paradigm employed may not fully replicate the complex stressors encountered by humans. Furthermore, the study utilized only intraperitoneal (i.p.) injection as the method of administration, which may differ from clinical drug delivery methods. Increasing the number of studies on multiple routes of administration, such as oral and transdermal patches, and comparing the antidepressant effects and pharmacokinetic profiles of PF under different modes of administration may provide some reference for the clinical application of the drug. Similarly, the depression model of rats established that the forced swimming trial (FST) was treated with PF (10 mg/kg) or fluoxetine (20 mg/kg) by gavage three times at 24 h, 5 h, and 1 h prior to the behavioral experiments, including the forced swimming and the open field trials. Both fluoxetine and PF could significantly shorten the immobilization time of the 5-min forced swim. In addition, the distance traveled in the open field did not significantly change in both groups compared to the normal control in the Open-Field Test (OFT) (Mu et al., 2020). Further studies revealed that PF was able to increase tolerance to stressors in rats by modulating the hyperactive HPA axis, exerting antidepressant effects similar to those of fluoxetine, as evidenced by the significant reduction of corticotropin-releasing hormone (CRH), ACTH, and CORT in plasma and hippocampus (Yan-xia et al., 2014). Meanwhile, the total glucosides of paeoniflorin (TGP) are active metabolites extracted from *Paeonia lactiflora* Pall. [Paeoniaceae, *Paeonia lactiflora root*], including PF, hydroxyl-paeoniflorin, paeonin, albiflorin, benzoylpaeoniflorin. Whereas, PF is the main active metabolite of TGP, which accounts for more than 40% of TGP (Zhang and Wei, 2020). Mao et al. found that TGP significantly inhibited the behavioral and biochemical changes in the chronic unpredictable mild stress (CUMS) mice, and could dose-dependently reduce the serum CORT levels in CUMS-exposed mice. The TGP may also exert an antidepressant-like effect by regulating the function of HPA (Mao et al., 2009a).

Numerous studies have found that prenatal stress (PS) increases the risk of neurological, endocrine, and metabolic disorders, which induces depression in offspring. To stressed offspring, PF (15, 30, and 60 mg/kg/day) administered for 28 consecutive days significantly increased sucrose intake and reduced immobility time as well as the total number of crossings, center crossings, rearing, and grooming in model rats. However, PF could restore the levels of ACTH, CRH, and COR in PS offspring (Li et al., 2020). For the ovariectomized and CUS-induced menopause depression model rats, PF could downregulate the serum levels of CRH, CORT, and ACTH, correct the hyperfunctioning of the HPA axis, which resulted in a significant improvement in the abnormal behaviors of the model rats (Huang et al., 2015). In summary, PF results suggest that PF could regulate mood disorders and exert antidepressant-like effects by modulating the disordered HPA axis function.

### 4.2 Monoaminergic nervous system

Most trial and clinical evidence have shown that monoaminergic neurotransmitters such as serotonin (5-HT), norepinephrine (NE), and dopamine (DA) have a wide range of biological activities and are important regulators of a series of physiological activities, such as mental activity, behavioral state, and emotion in the CNS (Nemeroff, 2002). Thus, physiological changes in abnormal monoaminergic neurotransmitters, including 5-HT, NE, and DA signal transduction could change receptor regulation and function, or impaired intracellular signal processing (Liu Y. et al., 2018), which trigger various emotional changes (Hamon and Blier, 2013). 5-HT, a biochemical messenger and modulator synthesized by decarboxylation of L-tryptophan, produces a variety of “state-dependent” behavioral responses to different stimuli, which have been shown to be useful in the treatment of anxiety and obsessive-compulsive disorder. The defects in the 5-HT system lead to disorders such as depression, phobias, obsessive-compulsive disorder, generalized anxiety disorder, and post-traumatic stress disorder (De-Miguel and Trueta, 2005). The most common of the pathophysiologic hypotheses in depression is the monoamine hypothesis, which posits that alterations in monoamine neurotransmitters are responsible for the pathogenesis of depression, such as decreased concentrations, abnormal function, and defective transmission across the synaptic gaps in the brain (Hirschfeld, 2000). Evidence for this hypothesis comes mainly from clinical observations and animal experiments. Reserpine, the antihypertensive drug, has been shown to deplete central stores of monoamines, which can trigger depressive-like manifestations such as bradykinesia and sedation (Delgado, 2000). In contrast, isoniazid could increase the concentration of NE and 5-HT in the brain by inhibiting monoamine oxidase, making the subject feel euphoric and active (Buda et al., 2022). The monoamine oxidase inhibitors (MAOIs) iproniazid also have antidepressant effects when used in patients with tuberculosis (Delgado, 2000). Therefore, increasing the monoaminergic neurotransmitter levels and enhancing the function of monoaminergic neurotransmitter systems are effective options for the treatment of depression. According to this hypothesis, various antidepressant drugs have been discovered, such as tricyclic antidepressants (TCAs) that block the reuptake of monoamines in presynaptic neurons, and MAOIs that prevent the breakdown of monoamines after reuptake and enhance neurotransmitters to exert antidepressant effects (Kessing et al., 2024).

The study found that the treatment of PF significantly attenuated the decrease of NA, DA, 5-HT, and metabolite 5-hydroxyindoleacetic acid (5-HIAA) in chronic CUS model mice, as well as the increase in the ratio of 5-HIAA/5-HT in the model (Qiu et al., 2013; Wang Jing-xia et al., 2010). Similarly, Intragastric administration of PF (10 mg/kg) could increase the levels of 5-HT and NE in plasma and hippocampus of the forced swimming test (FST) depression model rats (Mu et al., 2020). Reserpine is a vesicle reuptake inhibitor that irreversibly inhibits the vesicular uptake of monoamine neurotransmitters, including NA, DA, and 5-HT, which are metabolized and depleted by monoamine oxidase (MAO) inducing behavioral and physiological changes in animals (Ye et al., 2024). Based on the above principles, the rifampicin-antagonistic experimental model was the first established animal model of depression (Bourin et al., 1983). Intragastric administration of PF or TGP dose-dependently antagonized hypothermia, akinesia, and blepharoptosis in reserpine-induced mice, and reversed reserpine-induced decreases in monoamine transmitters such as NE, DA, and 5-HT in the brain, which showed an obvious anti-resensitization effect (Mao et al., 2008; Guang-zhi, 2012; Jin Shumei and Cui, 2013). These studies suggested that the antidepressant-like effect of PF or TGP may be realized by protecting monoamine neurotransmitters. In addition, PF or TGP could protect monoamine neurotransmitters by dose-dependently inhibiting the activities of MAO-A and MAO-B in the mouse brain monoamine oxidase (Mao et al., 2009b).

5-HT1A receptors, the largest class of 5-HT receptor subtypes, are mainly distributed in the frontal cortex, hippocampal area, lateral septal nucleus, and dorsal nucleus of the middle suture, which are closely associated with anxiety and depression (Sargent et al., 2010). 5-HT2A receptors are densely distributed in the hippocampal area, amygdala, prefrontal cortex, and olfactory cortex executive area, closely associated with suicide, depression, and schizophrenia (Papp et al., 1994). The mRNA and protein expression levels of the 5-HT1A receptor in the hypothalamus of menopausal depression rats by CUMS model were significantly lower than those of normal rats, while the mRNA and protein expression levels of the 5-HT2A receptor were significantly higher than those of normal rats. After gavage of PF (10 mg/kg) for 2 weeks, the mRNA and protein expression levels of the 5-HT1A receptor in the hypothalamus of the model rats were increased, while the expression level of the 5-HT2A receptor was decreased. These results indicate that PF can treat climacteric depression by adjusting the different 5-HT receptor subtypes in the hypothalamic region of rats (Huang et al., 2015). The model established by ovariectomy combined with long-term CUS in this study, while effectively simulating the physiological changes and stressors associated with menopause, still fails to capture the complex etiology and manifestations of human menopausal depression, and cannot fully replicate the realities of the human condition. In addition, although serum CRH, ACTH, CORT, and prefrontal cortex monoaminergic neurotransmitter levels, mRNA, and protein expression were measured, which confirmed the antidepressant effect of PF at the molecular level. On this basis, we suggest an in-depth exploration of the specific molecular targets where PF acts and the related signalling pathways. In addition, PF could activate the release of monoamines in the rodent brain, inhibit the reuptake of NA and 5-HT, and increase the content of DOPAC and 5-HIAA in the brain, which are the metabolites of 5-HT and DA (Lin et al., 2019; Qingwei, 2018).

### 4.3 Oxidative stress

Oxidative stress is caused by the overproduction of reactive oxygen species (ROS) or defective antioxidant systems, strongly associated with diseases such as cardiovascular disease, cancer, and diabetes (Poprac et al., 2017). In addition, ROS is widely recognized as a main cause of brain damage. Specifically, the brain effectively regulates oxygen consumption and redox-generating capacity by neutralizing the deleterious effects of ROS production through the antioxidant system, under normal physiological conditions. When this regulatory system is dysregulated, excessive ROS will cause oxidative damage to a series of biomolecules such as DNA, proteins, and lipids, and even lead to functional decline (Patel, 2016). Therefore, oxidative stress may be associated with the development of a range of neurodegenerative diseases or mental disorders, such as Alzheimer’s disease, Parkinson’s disease, cerebrovascular disease, attention deficit hyperactivity disorder, schizophrenia, and autism spectrum disorders (Vavakova et al., 2015). Most studies have shown that oxidative stress is closely related to depression. The depressed patients have elevated levels of ROS, reactive nitrogen species (RNS) (Suzuki and Colasanti, 2001; Dhir and Kulkarni, 2011; Maes et al., 2011), and reduced activity of the antioxidant glutathione (GSH) in postmortem brain samples (Gawryluk et al., 2011). In addition, the expression of the enzymes involved in ROS production (xanthine oxidase and monoamine oxidase) was increased in depressed patients. For example, xanthine oxidase (XO), which catalyzes the oxidation of xanthine to produce superoxide and hydrogen peroxide, has been observed to elevate levels in the serum of depressed patients and in the thalamic region of post-mortem depressed patients (Michel et al., 2010). MAO levels, of which the by-products, such as hydrogen peroxide, leading to excessive production of ROS, are higher in depressed and postpartum depressed patients than in nondepressed subjects (Sacher et al., 2015), resulting in neuronal apoptosis and mitochondrial dysfunction. Therefore, anti-oxidative stress may be an effective strategy for the treatment of depression.

**FIGURE 3:**
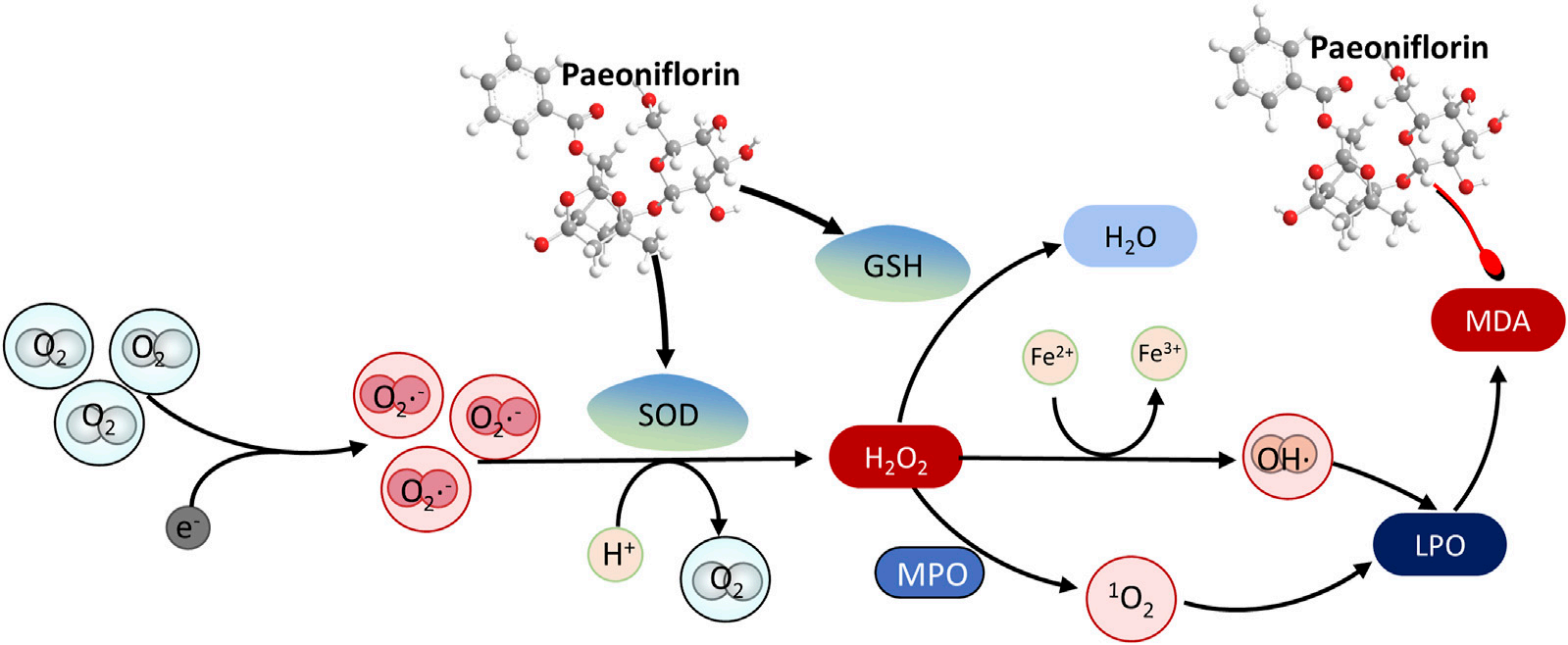
The antioxidant mechanism of paeoniflorin in depression by promoting the degradation of peroxides. The red arrow represents the inhibition of the protein, and the black arrow represents the promotion of the protein.

Numerous studies have demonstrated that PF could exert neuroprotective effects by inhibiting oxidative stress (Wang X. et al., 2021) (Figure 3). Superoxide dismutase (SOD) is an important antioxidant enzyme that catalyzes the breakdown of superoxide into oxygen and hydrogen peroxide, which is degraded by catalase under physiological conditions (Sies, 2015). In addition, the increase of ROS could lead to lipid peroxidation of the cell membrane to produce a large amount of malondialdehyde (MDA), a product of oxidative stress. Clinical studies have reported higher serum levels of MDA in patients with major depression compared with controls (Sies, 2015). After multiple intragastric administrations of PF, the plasma SOD level of the depression rats in the FST model was increased, and the plasma MDA level was decreased (Mu et al., 2020). PF significantly shortened the 5-min swimming immobility time of the rats in the FST model and increased the 5-min moving distance in the open-field trials, showing an antidepressant effect similar to fluoxetine. These studies, however, still exhibit certain deficiencies in their design. Firstly, the use of fluoxetine exclusively as a positive control in the antidepressant group restricts a comprehensive evaluation of the advantages and disadvantages of PF in comparison to other similar medications. Additionally, the absence of a detailed randomization methodology for the experimental subgroups may compromise the results. Furthermore, the experimental design featured a limited range of behavioral tests, encompassing only the forced swimming test and the open field test, and was conducted at only a few specific time points, which hindered the ability to observe the effects of PF across different time periods. In CORT- or glutamate-treated PC12 cells, PF increased the cell viability, the levels of GSH, the SOD and catalase activities, meanwhile, decreased intracellular reactive ROS and MDA levels in a dose-dependent manner (Mao et al., 2012a; Mao et al., 2011a; Mao et al., 2010). In addition, long-term PF (15 mg/kg and 30 mg/kg, i.p.) treatment of Aβ (1–42) (1 μg/μL) in rats restored the decreased activities of SOD and catalase, increased the level of MDA and the content of reduced GSH, which suggests that the PF can exert neuroprotective effects by alleviating oxidative stress (Lan et al., 2013; Zhong et al., 2009). Furthermore, Mao QQ et al. found that TGP (80 and 160 mg/kg) treatment in CUS mice dose-dependently reduced GSH depletion and MDA production, which further suggests that the antidepressant-like activity of TGP may be mediated by attenuating oxidative stress in the mouse brain (Mao et al., 2009b).

### 4.4 Apoptosis

Apoptosis, a form of programmed cell death, plays a critical role in tissue homeostasis, immune cell renewal, and neuronal development, which prevents the damage of surrounding tissues by timely eliminating senescent or damaged cells in the body (McKernan et al., 2009). Apoptosis is a tightly regulated and energy-dependent process, characterized by cytoplasmic shrinkage, chromatin condensation, nuclear pyknosis, ordered DNA fragmentation, cell rounding, and membrane blistering. Cells eventually form the membrane-bound vesicles called “apoptotic vesicles”, which could be phagocytosed by neighboring cells. Most studies have shown that neuronal apoptosis plays a crucial role in most CNS diseases. For instance, excessive apoptosis of a series of neurons can lead to neurodegeneration such as Alzheimer’s disease, Parkinson’s disease, and Huntington’s disease (Su et al., 1994; Jenner and Olanow, 1998; Kim et al., 1999). In addition, apoptosis may also be caused by stress (Lucassen et al., 2006). The study showed numbers of apoptotic and necrotic cells were observed in depressed mice with chronic mild stress (CMS) (Tian et al., 2019). Kosten et al. found that exposure to unpredictable stress decreased Bcl-2 mRNA in limbic structures of the brain and frontal cortex, and Bcl-xL mRNA in the hippocampus (Kosten et al., 2008). Meanwhile, the Administration of alanylcyclopropionate, reboxetine, and fluoxetine upregulated Bcl-2 mRNA levels and also increased Bcl-xL mRNA expression. Moreover, Bcl-2 expression in the hippocampus was increased after 14 days of administration of citalopram, promethazine, and amitriptyline (Kosten et al., 2008), which suggests that potential antidepressants could be developed through an anti-apoptotic mechanism.

**FIGURE 4:**
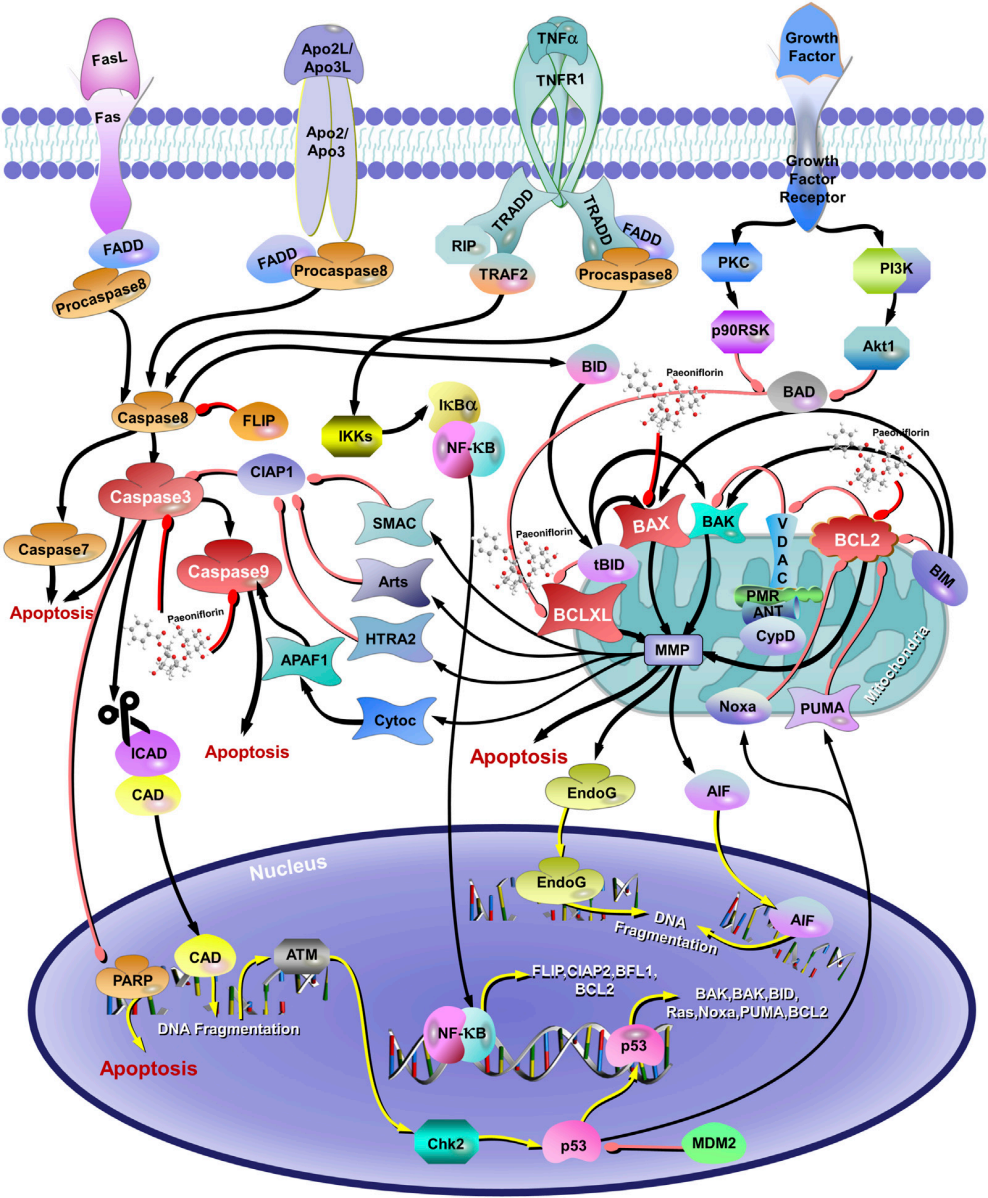
The Antiapoptotic mechanisms of paeoniflorin in depression. The red arrows represent the inhibition of proteins and the black arrows represent the promotion of proteins.

Studies have shown that PF, the natural plant metabolite from the treasure trove of nature, has surprising neuroprotective effects, inhibiting excessive neuronal apoptosis and exhibiting good therapeutic effects on neurodegenerative diseases (AD, Parkinson’s disease, etc.) and mental disorders (anxiety, depression, etc.) (Zhong et al., 2009; Zhai et al., 2019; Li et al., 2014; Zhang et al., 2017; Cong et al., 2019; Zhang et al., 2015; Wang et al., 2013b; Wu et al., 2013; Sun et al., 2012; Zheng et al., 2016). The accumulation of glutamate (Glu) in the synaptic cleft could produce excitatory neurotoxicity that may contribute to depression, anxiety, post-traumatic stress disorder, schizophrenia, cognitive impairment, and other psychiatric disorders (Mehta et al., 2013). Wang X et al. suggest that PF significantly improves the Glu-induced decrease in SH-SY5Y cell viability in human neuroblastoma cells by affecting the expression of Bax/Bcl2, cleaved caspase-3, and cleaved caspase-9, suggesting that PF significantly reduces cell apoptosis and exerts neuroprotective effects through the Bax/Bcl2 pathway (Wang X. et al., 2021) (Figure 4). Moreover, PF exhibited a suppressive effect on apoptosis by attenuating mitochondrial membrane potential, promoting cytochrome c release, and counteracting the upregulation of caspase-3 and caspase-9 in the case of Aβ 25–35 induced PC12 cell damage (Li et al., 2014). Treatment with 200 μM CORT resulted in apoptosis of PC12 cells for 48 h. Interestingly, the treatment of TGP protected PC12 cells against CORT-induced toxicity in a dose-dependent manner, which was associated with the inhibition of caspase-3 activity involved in the mitochondrial pathway and the upregulation of the bcl-2/bax mRNA ratio. Meanwhile, TGP in mice also produced similar antidepressant effects (Mao et al., 2009c). Similarly, XiaoyaoSan, a characteristic Chinese traditional formula, reduced chronic stress-induced anxiety and depression behaviors in mice. PF, one of the main active plant metabolites in XYS, could protect primary neurons from CORT-induced neurotoxicity and reverse neuronal apoptosis caused by miR-200a-3p and miR-200b-3p overexpression (Yuan et al., 2022). It is important to note that XiaoyaoSan, as a Chinese medicine formula, has a complex composition that includes various plant metabolites beyond PF. This study focused solely on miR-200a/b-3p and PF, neglecting the potential roles and interrelationships of other miRNAs and metabolites within the formula. Furthermore, the use of a stereotactic microinjection method to investigate the role of miR-200a/b-3p in stress behavior may pose a risk of damaging rat brain tissue, which could subsequently interfere with the experimental results. The above studies suggest that PF may exert antidepressant effects through an anti-apoptotic molecular mechanism.

### 4.5 Calcium homeostasis and calcium signaling pathways

Calcium signaling regulates a range of neuronal activities by controlling the release of multiple neurotransmitters (Bezprozvanny and Mattson, 2008; Bergantin et al., 2013). Dysregulation of calcium signaling, such as excessive Ca^2+^ influx, may be associated with aging, Alzheimer’s disease, and major depression (Bergantin and Caricati-Neto, 2016). The abnormality of serum calcium level may be related to cognitive impairment in patients with depression (Grutzner et al., 2018). Normally, the intracellular Ca^2+^ concentration is maintained at a stable low level. Excessive Ca^2+^ influx may over-activate the Ca^2+^/CaM/CaMKII signaling pathway, impair neuronal activity, and induce depression when the homeostasis is changed. In addition, previous studies have found that the development of depression leads to neuronal death in the hippocampus and limbic brain. And the dysregulation of intracellular Ca^2+^ homeostasis seems to be closely related to this abnormal neuronal death (Duman, 2004; Bergantin, 2020). Therefore, maintaining normal Ca^2+^ homeostasis and safeguarding the proper regulation of calcium signaling pathways is one of the mechanisms to protect the nervous system.

Calbindin-D28K, one of the major calcium-binding proteins in the brain, could maintain intracellular Ca^2+^ homeostasis by binding excess Ca^2+^ to protect normal neuronal activation and function and inhibit neuronal apoptosis caused by intracellular Ca^2+^ overload (Carter et al., 2008; Meng et al., 2007). Experiments showed that PF could reverse the decrease of Calbindin-D28K mRNA level and the increase of Ca^2+^ concentration in PC12 cells induced by Glu, suggesting that the neuroprotective effect of PF is related to the inhibition of intracellular Ca^2+^ overload (Mao et al., 2010; Mao et al., 2011b). This result may be one of the pathways of antidepressant activity of PF *in vivo*. Meanwhile, PF treatment widely observed in other *in vitro* and animal trials could significantly block intracellular calcium influx caused by adverse stimuli such as methyl-4 phenylpyridine ion (MPP^+^) (Wang et al., 2013b), Aβ(1–42) oligomer (Zhong et al., 2009), and Glu (Wang X. et al., 2021; Wang et al., 2013c), which improve intracellular calcium homeostasis to play a neuroprotective role. In addition, previous studies have shown that the Ca^2+^/CaMKI/CREB signaling pathway plays an important role in intracellular signaling pathways involved in cell proliferation, cell survival, inflammation, and metabolism (Meng et al., 2007; Valera et al., 2008; Zhang et al., 2012). Calcium/calmodulin-dependent pathways may be overactivated to irreversible cellular damagewhen calcium is overloaded in neurons. PF reversed the significant reduction of p-CaMKII and p-CREB and regulated the expression of downstream proteins in the Middle Cerebral Artery Occlusion(MCAO) model and N-methyl-D-aspartic acid receptor(NMDA) induced excitatory toxicity model of primary hippocampal neurons, including Bax, Bcl2, Bad, and Caspase3, along the Ca^2+^/CaMKI/CREB signaling pathway (Zhang et al., 2017). Song et al. found that the effect of PF on the Ca^2+^/CaMKI/CREB signaling pathway may be achieved by regulating the current density of voltage-gated Ca^2+^ channel Cav1.2 (Song et al., 2017). Another study found that PF could significantly inhibit Glu-induced CaMKII over-expression and prevent intracellular calcium overload in PC12 cells (Wang et al., 2013c), thus playing an important role in the treatment and remission of affective disorders.

### 4.6 Neuroinflammation

Recently, clinical and preclinical evidence suggestthat neuroinflammation is an important factor in major depressive disorder. Studies using positron emission computed tomography (PET) imaging and 18 kDa translocator protein (TSPO) as a microglia biomarker have demonstrated neuroinflammation in several brain regions in depressed patients (Li et al., 2018; Setiawan et al., 2018). The research in animal models has also revealed the release of proinflammatory factors and activation of microglia in the animal brain, showing signs of anxiety and depression (Munshi et al., 2020; Wang Y. L. et al., 2018). The persistent sympathetic and parasympathetic under-activity in chronic stress situations and major depressive disorder (MDD) increase catecholamine levels and decreases acetylcholine levels, which increases levels of pro-inflammatory cytokines (TNF-α, IL-1β, IL-6, and IL-18, among others), which explains that depression-like symptoms can be induced directly by proinflammatory cytokines (Won et al., 2021). Moreover, proinflammatory cytokines could activate the kynurenine pathway, leading to the increase of neurotoxic metabolites, including 3-hydroxykynurenine, 3-hydroxy-anthranilic acid, and quinolinic acid, to cause brain damage (Kim and Won, 2017). Meanwhile, researchers have found that the activation of microglia by stress stimulation releases a large number of proinflammatory cytokines, which could destroy the neuroprotective mechanisms in the brain, impair neuroplasticity, and inhibit adult hippocampal neurogenesis, leading to the occurrence of depression-related symptoms (McKim et al., 2016).

**FIGURE 5:**
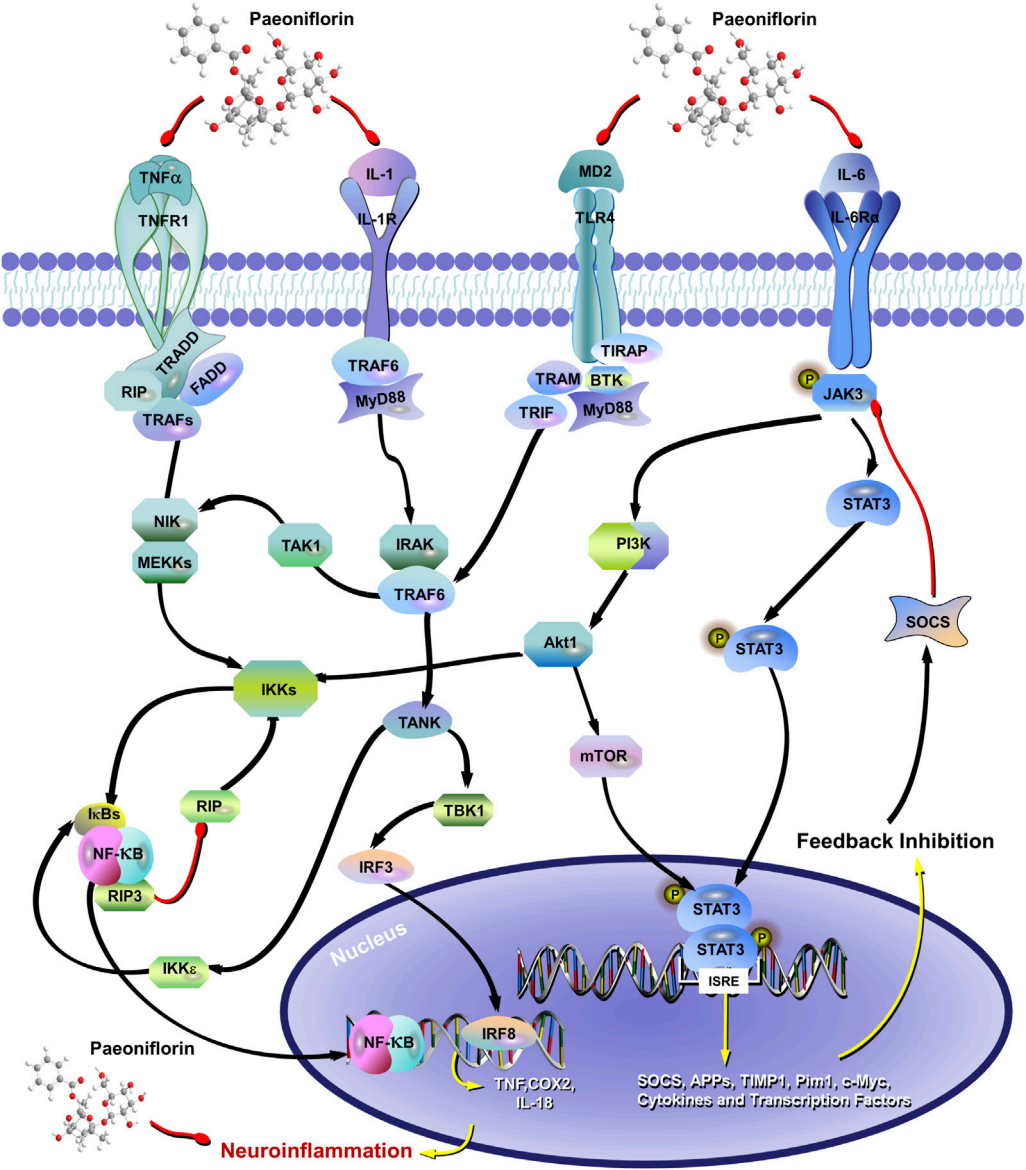
The mechanism of paeoniflorin inhibiting neuronal inflammation involves the inhibition of IL-1, IL-6, and TNF-α Expression, and affects the TLR4/NF-κB/NLRP3 signaling pathway. The red arrow represents the inhibition of the protein, and the black arrow represents the promotion of the protein.

The researchers have recently found that PF significantly inhibits the neuroinflammatory response by decreasing the over-activation of astrocytes (AST) and microglia, as well as the expression of pro-inflammatory cytokines such as IL-1β, IL-6 and TNF-α (Zhou et al., 2019; Liu H. et al., 2015; Nam et al., 2013) (Figure 5). PF prevented the upregulation of pro-inflammatory mediators (TNF-α, IL-1b, iNOS, COX2, and 5-LOX) in plasma and brain to the sustained activation of hippocampal AST and microglia caused by chronic cerebral insufficiency of cerebral perfusion or cerebral ischemia, suggesting that PF exerts delayed protective effects in ischemia-injured rats by inhibiting the peripheral and cerebral tissue inflammatory responses mediated in MAPKs/NF-kB (Guo et al., 2012; Liu J. et al., 2006). TLR4/NF-κB/NLRP3 signaling has been shown to regulate the inflammatory response of microglia, of which the activation promotes the over-expression and over-release of proinflammatory cytokines, leading to neuronal damage (Zhong et al., 2019). Cheng et al. found that PF can reduce the release of pro-inflammatory cytokines by regulating TLR4/NF-κB/NLRP3 signaling, thus reducing the damage of cytokines to neurons and reversing LPS-induced depression-like behavior in mice (Cheng et al., 2021). The study found obvious changes in inflammation in the amygdala for 4 weeks after subcutaneous injection of Interferon-α 15 × 10^6^ IU/kg, as modeled by depression-inducing mice. Interestingly, the trial after 4 weeks of pretreatment with PF (20 mg/kg or 40 mg/kg) reversed the depression behavior of mice, and the abnormal level of inflammatory cytokines in serum, medial prefrontal cortex(mPFC), ventral hippocampus (vHi) and the amygdala, including IL - 6, IL - 1β, TNF-α, IL - 9, IL - 10, IL - 12 and monocyte chemotactic protein 1 (Li et al., 2017). However, the experiment only established two PF dose groups, specifically 20 mg/kg and 40 mg/kg, which limits the ability to ascertain the optimal effective dose of PF and its dose-effect relationship. Furthermore, while PF was observed to reverse the increase in microglia density and reduce the levels of inflammatory factors, the specific molecular pathways through which PF influences microglia in the context of neuroinflammation and depressive-like behaviors have not been thoroughly investigated. Furthermore, recent studies have found that PF reduces neuroinflammation by inhibiting Casp-11-dependent pyroptosis signaling induced by the overactivation of hippocampal microglia in reserpine-treated mice, representing a novel mechanism by which PF attenuates depression (Tian et al., 2021).

### 4.7 Mitochondrial dysfunction

Mitochondria, the energy factory of eukaryotic cells, produce energy through the metabolism of lipids, steroids, and proteins, which play a key role in maintaining cellular stability by regulating Ca^2+^ levels, maintaining ROS levels, and regulating apoptosis (Nunnari and Suomalainen, 2012). Mitochondrial biosynthesis occurs more rapidly during neuronal development because neuronal differentiation requires an increase in the mitochondrial genome and mitochondrial proteins (Calingasan et al., 2008; Cuperfain et al., 2018; Kasahara and Kato, 2018). Therefore, mitochondrial dysfunction prevents cells from meeting their energy requirements and involves impairment of neuronal communication, cellular resilience, and hippocampal neurogenesis, which leads to mood and psychiatric disorders (Perkins et al., 1997; Nagashima et al., 2020). Most studies have shown that mitochondrial dysfunction in brain regions may be closely related to the development of depression. For example, brain mitochondrial dysfunction and ultrastructural damage have been reported in mouse models of depression (Gong et al., 2011). 54% of patients were also found to exhibit depressive symptoms in a study of the prevalence of psychiatric comorbidities in subjects with mitochondrial cytopathies (Feng et al., 2020), suggesting that targeting mechanisms of mitochondrial dysfunction may be a possibility for developing new treatments for depression.

Studies have shown that PF could exert neuroprotective effects by preventing mitochondrial dysfunction. PF could significantly improve the viability of SH-SY5Y cells and human neuroblastoma cells damaged by Glu or Ab25-35, and significantly inhibit the increase of mitochondrial membrane potential and calcium concentration to protect SH-SY5Y cells from Glu-induced excitatory neurotoxicity (Wang X. et al., 2021; Li et al., 2014). Daily administration of PF (10 mg/kg once a day) for 21 days was able to significantly ameliorate the cognitive dysfunction for streptozotocin (STZ)-induced in mice model, This research has demonstrated that PF may be related to the significant attenuation of the STZ-induced mitochondrial dysfunction, including a significant increase in cytochrome C oxidase activity and ATP synthesis to significantly restore the function in hippocampal area and cerebral cortex (Wang D. et al., 2018). Moreover, PF can regulate mitochondrial membrane potential and maintain mitochondrial membrane integrity to attenuate or restore PC12 cell injury induced by unfavorable stimuli such as Aβ25-35, MPP^+^, Glu, and CORT (Li et al., 2014; Wang et al., 2013b; Sun et al., 2012; Zheng et al., 2016; Mao et al., 2009c). Further studies revealed that this preventive-protective effect by PF might be achieved by inhibiting the MKK4-JNK signaling pathway (Cong et al., 2019).

### 4.8 Autophagy

Autophagy, a process of cellular waste removal and regeneration, transports cytosolic components such as proteins and organelles to lysosomes for degradation and recycling to provide a constitutive mechanism for the turnover and recruitment of cellular components (Xiao et al., 2018; Miranda et al., 2018). In the CNS, autophagy is involved in cell surveillance, neuroinflammation, and neuroplasticity. The investigators found that neurons are vulnerable to autophagy defects and depend heavily on the level of autophagy composition for survival (Hara et al., 2006; Nishiyama et al., 2007). Several preclinical and clinical studies have shown that dysfunction of cellular autophagy may be an important factor in the occurrence and progression of psychiatric disorders such as depression (Komatsu et al., 2006; Nouri et al., 2020; Zhao et al., 2017). For example, autophagy biomarkers, such as the LC3II/LC3I ratio and BECN1, are significantly reduced in animal models of depression. Notably, PF can exert neuronal protection by modulating the autophagy pathway (Jiang P. et al., 2017). PF can have a significant protective effect against acidosis or MPP + -induced injury in PC12 cells; further research suggested that PF improved acidosis-induced activation of acid-sensing ion channels (ASIC).

Meanwhile, PF weakened the autophagy inhibition induced by MPP^+^ and significantly upregulated autophagy and the ubiquitin-proteasome pathway, which prevents α-synuclein accumulation(α-SYN) of synaptic nucleoproteins to reduce cell damage (Sun et al., 2011; Cai et al., 2015; Cao et al., 2010). α-SYN is a characteristic marker of all types of PD, which can be degraded by the ubiquitin-proteasome system (UPS) and autophagy-lysosomal pathway (ALP) (Kaushik and Cuervo, 2012). In another study, systemic administration of PF in a 6-hydroxydopamine (6-OHDA) -induced PD rat model showed that PF enhanced the autophagic degradation of α-SYN to protect DA neurons from 6-OHDA-induced neurotoxicity. Further Western blot results showed that PF could significantly reduce the level of ASIC1a, suggesting that PF may play a neuroprotective role by inhibiting the activation of ASIC, especially ASIC1a (Gu et al., 2016).

### 4.9 Brain-derived neurotrophic factor

Brain-derived neurotrophic factor (BDNF), a key mediator of activity-dependent neuronal plasticity in the brain (Park and Poo, 2013), has a major impact on neuronal morphology, survival, and differentiation, increasing synapse sprouting and synaptic stability, and facilitating long-duration strengthening (Zagrebelsky and Korte, 2014). BDNF synthesis occurs predominantly in regions involved in emotional and cognitive functions, such as the hippocampus and frontal regions (Phillips, 2017). BDNF could bind and activate tropomyosin receptor kinase B (TrkB) to regulate the different cellular processes for the development and maintenance of normal brain function. In addition, several lines of evidence suggest that BDNF/TrkB signaling is involved with adult neurogenesis in the hippocampus and has differential effects on the dentate gyrus (DG) and subventricular zone (SVZ) (Colucci-D'Amato et al., 2020). Numerous studies have confirmed that BDNF is one of the important biomarkers of depression. The low levels of BDNF are associated with reduced synaptic plasticity and neuronal atrophy, which is consistent with the neurogenic hypothesis of depression. Several autopsy studies have demonstrated decreased BDNF expression in the hippocampus and prefrontal cortex of depressed patients (Dwivedi et al., 2003). In addition, significantly elevated BDNF levels were detected in the serum of patients treated with antidepressants. This suggests that BDNF is not only a marker of disease but also a potential predictor of antidepressant efficacy. Taking BDNF signaling as a breakthrough point would be a potential direction for the development of antidepressants.

Fortunately, a large number of studies have found that the natural plant metabolite PF could upregulate the expression of plasma BDNF and BDNF mRNA in the hippocampus and frontal cortex (Mu et al., 2020; Mao et al., 2009a; Qingwei, 2018; Mao et al., 2012b), as well as postsynaptic density protein 95 (PSD 95) in model animals with mood disorders (Liu S. C. et al., 2019), thereby preventing CUMS-induced synaptic plasticity defects and providing neuroprotection in animal models of mood disorders. Previous studies suggested that BDNF combined with p-CREB is the main regulator of neurogenesis and emotion regulation, which may be closely related to neural plasticity (Sasaki and Yoshizaki, 2015; Orlovsky et al., 2014). Hu et al. found that PF therapy plays a positive role in neural and emotional regulation by reversing the decreased expression of BDNF and p-CREB in the hippocampus caused by the MCAO and CUMS model (Hu et al., 2019). In addition, PF also significantly increased the levels of other neurotrophic factors, such as NGF protein and mRNA, in the frontal cortex of CUMS rats. Further study found that the treatment of PF not only significantly enhanced the protein expression and gene transcription of BDNF in CUMS rats, but also activated the expression of TrkB, a high-affinity receptor for BDNF, which promoted the proliferation of neural stem cells, differentiation into AST, and neurogenesis in the DG of the hippocampus in stressed rats. This result suggests that PF may play the role of an antidepressant through the BDNF/TrkB signaling pathway (Chen et al., 2019). Meanwhile, Chen et al. used PF to treat the withdrawal hormone simulated pregnancy (HSP) in a Postpartum depression (PPD) rat model and found that PF improved PPD symptoms by promoting the activation of the transporter TspO and BDNF/mTOR pathways in PPD rats, proving that PF may be an effective anti-PPD and anti-depression drug (Chen J. et al., 2022).

## 5 Safety overview

Currently, toxicological studies on PF are still very limited. Most of the studies have investigated the toxicity of PF at the cellular level. In general, PF is a low-toxicity natural plant metabolite. The safety results of PF still vary in different cell types. For mouse thymocytes, PF did not show cytotoxicity at concentrations of 0–1000 μg/mL (Li et al., 2007). Two studies reported that PF showed a favorable safety profile against U937 cells, the human myelomonocytic cell line, which did not exhibit cytotoxicity even at 640 μg/mL (Salunga et al., 2007; Jin et al., 2011). However, our study found that PF at 400 μg/mL exhibited some growth inhibition against BEAS-2B cells, the human normal lung epithelial cells (Hou et al., 2024). Meanwhile, PF also exhibited some toxicity with HaCat cells in a concentration-dependent manner (Wang D. et al., 2022). The safety studies of PF with animal models are still scarce *in vivo*. PF was found to inhibit the hatching rate of zebrafish at high concentrations (100 and 200 μg/mL) (Rao et al., 2024). Some clinical trials of formulas containing PF have reported the safety evaluation. The pharmacokinetic trial involving Chinese volunteers showed that no adverse events (AEs) and serious adverse events (SAEs) were observed in the investigators who received a single intravenous infusion of Huoxue-Tongluo lyophilized powder for injection (HTLPI) containing PF (Li et al., 2016). Interestingly, one male subject experienced elevated transaminase levels with multiple dose infusions that recovered after 2 weeks (Li et al., 2016). Another clinical trial conducted on Chinese healthy volunteers reported that injections containing PF can cause AEs such as dizziness and diarrhea, but no SAEs occurred (Chen et al., 2013). Indeed, both clinical trials used formula preparations containing PF, and the subjects were all from China, which may interfere with the trial results. These clinical results can only provide a reference for the safety evaluation of PF.

In conclusion, although the above studies reported some safety results of PF, most of them were not systematically investigated. Meanwhile, the long-term evaluative safety assessment of PF should be emphasized by researchers. Particularly, how to select reference reagents and placebos is also a challenge for the clinical design of natural plant metabolites, which is necessary for the pharmacological research of PF.

## 6 Traditional Chinese medicine formula

In the application of traditional Chinese medicine, certain formulas containing PF have demonstrated notable efficacy in treating depression, such as Xiaoyao San, Chaihu Shugan San, and Sini San. These formulas, which include PF as one of their active ingredients, exert their effects through multiple targets and pathways, involving the regulation of neural pathways, neurotransmitters, synaptic plasticity, the neuroendocrine system, and the immune system. Although PF may not be the decisive factor in the prescription, TCM formulas containing PF still hold significant value in the treatment of depression. TCM formulas are composed of multiple botanical drugs that are synergistically combined, with each botanical drug containing various plant metabolites that interact to produce therapeutic effects. The following section will summarize the applications and molecular mechanisms of several classic TCM formulas containing PF in the treatment of depression.

Xiaoyaosan (XYS) is a classic TCM formula originating from the “Taiping Hui Min He Ji Ju Fang”, which is composed of *Bupleurum chinense* DC. [Apiaceae, *Bupleurum chinense root*], *Angelica sinensis* (Oliv.) Diels [Apiaceae, *A. sinensis radix et rhizoma*], *Paeonia lactiflora* Pall. [Paeoniaceae, *Paeonia lactiflora root*], *Atractylodes macrocephala* Koidz. [Asteraceae, *Atractylodes macrocephala rhizoma et root*], *Wolfiporia cocos* (F.A. Wolf) Ryvarden & Gilb. [Polyporaceae, *W. cocos sclerotium*], *Glycyrrhiza uralensis* Fisch. ex DC. [Fabaceae, *Glycyrrhiza uralensis radix et rhizoma*], *Mentha canadensis* L. [Lamiaceae, *M. canadensis leaves*], and *Zingiber officinale* Roscoe [Zingiberaceae, *Z. officinale rhizoma et root*], with a recommended ratio of 6:6:6:6:6:3:2:2. Its active metabolites include PF, bupleurum saponins A/C/D, ferulic acid, ligustilide, atractylodes lactone I/II/III, paeonia lactiflora glycoside, glycyrrhiza glycoside, glycyrrhetic acid, and poria acid (Lu et al., 2018). This formula has a history of over 2,000 years in China and is widely employed to treat various types of depression due to its liver-soothing, depression-relieving, spleen-nourishing, and blood-nourishing effects (Feng et al., 2016; Li et al., 2022). Recent studies have demonstrated that XYS exerts its antidepressant effects through multi-target regulation, involving the nervous, endocrine, and immune systems (Ma et al., 2019) (Tables 1, 2). For example, XYS can reduce the expression of miR-200a/b-3p in the prefrontal cortex induced by chronic stress, regulating the miR-200a/b-3p/NR3c1 signaling pathway. Further studies have found that PF, its main metabolite, inhibits miR-200a/b-3p-mediated neuronal apoptosis (Yuan et al., 2022). Additionally, XYS can reverse CORT elevation in the HPA axis of CUMS model rats, upregulate glial fibrillary acidic protein (GFAP) expression in the hippocampus, influence astrocyte (AST) activity, downregulate the NMDA receptor NR2B subunit level in the hippocampus, and improve depressive-like behavior (Liu T. et al., 2020). Studies on APJ receptors indicate that XYS can upregulate hypothalamic apelin levels and downregulate APJ levels, resulting in antidepressant behavioral improvements comparable to those of fluoxetine (Yan et al., 2018). Additionally, XYS can also increase microtubule-associated protein 2 (MAP2) expression in the CA1 region of the hippocampus in CUMS rats, enhance NR2B and PI3K expression to regulate the NR2B and PI3K/Akt signaling pathways, and alleviate Glu-induced neuronal damage (Zhou et al., 2021). Interestingly, XYS may exert its antidepressant effects by regulating gut microbiota composition and restoring abnormal levels of cecal metabolites (Lv et al., 2021).

**TABLE 1:** Antidepressant Mechanisms of Formulas *in vivo*. (XYS: Xiaoyaosan; CSS: Chaihu-Shugan-San; SNS: Si-Ni-San).

Formula	Subjects	Control group		Experimental group	Time	Mechanisms	Source
		Model group	Positive drug control group				
XYS	Chronic immobilization stress (CIS) SD rats	Distilled water	Fluoxetine (1.76 mg//kg/d)	XYS decoction (3.854 g/kg/d)	21 days	decreased concentrations of nesfatin-1 (NES1) in the serum and paraventricular nucleus, reduced expression levels of proopi-omelanocortin (POMC), oxytocin (OT), and melanocortin-4 receptor (MC4R) in the hypothalamus; regulate the NES1-OT-POMC neural pathway in the hypothalamus	Ma et al. (2019)
	CUMS SD rats	Deionized water	Fluoxetine (2.0 mg/kg/d)	XYS suspension (2.22 g/kg/d)	6 weeks	reduced the expression of miR-200a/b-3p and neuronal apoptosis in the prefrontal cortex (PFC); regulate miR-200a/b-3p/NR3C1 signaling in the PFC	Yuan et al. (2022)
	CUMS SD rats	Deionized water	Fluoxetine (2.0 mg/kg/d)	XYS suspension (2.224 g/kg/d)	42 days	reduced the expression of serum corticosterone (CORT) and hippocampus glutamate; improved the expression of NR2B (**↓**) and glial fibrillary acidic protein (GFAP ↑)in the hippocampus	Song et al. (2020)
	CUMS C57BL/6 J mice	Normal saline	Fluoxetine (2.6 mg/kg/d)	XYS decoction (0.25 g/kg/d)	21 days	increase the protein levels of GFAP, neuronal nuclear antigen (NeuN), excitatory amino acid transporters 1 and 2 (EAAT 1/2) in PFC	Liu et al. (2019b)
	CUMS SD rats	Distilled water	Fluoxetine (2 mg/kg/d)	XYS suspension (2.224 g/kg/d)	21 days	decrease the level of glutamate in the hippocampal CA1 region and serum CORT, increase the expression of MAP2, NR2B, phosphoinositide 3-kinase (PI3K) and the P-AKT/AKT ratio in the hippocampal CA1 region	Zhou et al. (2021)
	CUMS C57BL/6 mice	Physiological saline	Fluoxetine (2.6 mg/kg/d)	XYS suspension (0.254 g/kg/d)	21 days	regulate the expressions of the enzyme-glutathione peroxidase 4 (GPX4), ferritin heavy chain 1 (FTH1), long-chain acyl-CoA synthetase 4 (ACSL4), cyclo-oxygen-ase 2 (COX2), phoshaptidylethanolamine binding protein 1 (PEBP1), extracellular regulated protein kinases 1/2 (ERK1/2), GFAP and ionic calcium junction protein molecule-1 (IBA-1), and change the total iron and ferrous content in the hippocampus	Jiao et al. (2021)
	chronic restraint stress (CRS) SD rats	Normal saline	—	XYS suspension (2.224 g/kg/d)	21 days	improves synaptic survival and growth in the stratum, reduces adenosine A (2A) receptor (A2AR) activity and suppresses hyper-activation of striatal microglia	Zhu et al. (2022)
	CUMS C57BL/6J mice	Normal saline	Fluoxetine (2.6 mg/kg/d)	XYS decoction (0.25 g/kg/d)	28 days	reduce the depressive-like behavior of mice and inhibit the expression of the inflammationrelated receptor of advanced glycation protein end product (RAGE) and mRNA in the cingulate gyrus (Cg), and increase the functional connectivity (FC) of the Cg in mice	Yan et al. (2021)
	CUMS C57BL/6J mice	Distilled water	Fluoxetine (2.6 mg/kg/d)	XYS suspension (0.658 g/kg/d)	21 days	regulate the autophagy in hypothalamic neurons, improve the expression of LC3 (↑), p62 (↓), and glucose transporter-4 (GLUT4 ↑)	Yang et al. (2022)
CSS	Post-stroke depression (PSD) SD rats	Mod	Empty virus; GSK3β overexpressing virus; JAK-STAT3 inhibitor (50 μM)	CSS suspension (4.4 g/kg)	21 days	decrease interleukin (IL)-1, IL-6, tumor necrosis factor (TNF)-α, and increase IL-10, improve the level of STAT3(↓), PTEN(↓), and GSK3β(↑), inhibite neuroinflammation by regulating microglia polarization through activation of the JAK/STAT3-GSK3β/PTEN/Akt pathway	Fan et al. (2023)
	Perimenopausal syndrome (PMS) + CUMS SD rats	Sham group; PMS group; PMS + CUMS group. (Normal saline)	—	CSS decoction (3.31 g/kg/d)	21 days	improve the brain functional connectivity between the hippocampus and other brain regions, improve the concentrations of citrate, isocitrate and guanosine triphosphate (GTP) among the metabolites in the hippocampal tricarboxylic acid cycle	Huang et al. (2024)
	CUMS C57BL/6 mice	Distilled water	—	CSS suspension (19.5 g/kg/d)	42 days	induce angiogenesis, increase Silent information regulator protein 1 (SIRT1) expression, and decreased Forkhead box O1 (FOXO1) expression in the hippocampus, upregulate vascular endothelial growth factor (VEGF) and BDNF expressions in the hippocampus and brain microvascular endothelial cells (BMVECs) supernatants	Zhang et al. (2022)
	perimenopausal syndrome (PMS) + CUMS SD rats	Sham group; PMS group; PMS + CUMS group. (Normal saline)	—	CSS decoction (1 g/kg/d)	21 days	upregulate the expressions of PI3K and Akt, affect PI3K/Akt signalling pathway	Chen et al. (2022b)
	restraint stress (RS) C57BL/6 mice	Normal saline	PC (1 mg/kg buspirone)	CSS decoction (1 g/kg/d)	5 days	suppresse the activation of NF-κB and expression of interleukin (IL)-6, and increase the expression of BDNF, suppresse IL-6 and CORT level in the blood and IL-6 expression and myeloperoxidase activity in the colon; decrease the γ-Proteobacteria population, increase Lactobacillaceae, Prevotellaceae, and AC160630_f populations	Han et al. (2021)
	CUMS C57BL/6 mice	Normal saline	—	CSS suspension (2.7 g/kg/d)	42 days	increase phosphorylated (p) PI3K/PI3K and pAKT/AKT levels and decrease the pGSK3β/GSK3β level in the hippocampus	Zhang et al. (2021b)
	CUMS SD rats	Normal saline	Fluoxetine (1.8 g/kg/d)	CSS decoction (5.9 or 11.8 g/kg/d)	—	increase the expression of BDNF, p-CREB/CREB, p-ERK/ERK, and BDNF mRNA, CREB mRNA, and ERK mRNA in the hippocampus and frontal cortex; regulate the BDNF/ERK/CREB signaling pathway	Yan et al. (2020)
SNS	Kunming mice	Normal saline	Fluoxetine (20 mg/kg)	SNS decoction (325, 650, and 1300 mg/kg)	—	decrease serum CORT levels, elevated serotonin (5-HT), norepinephrine (NE), and dopamine (DA) levels	Yi et al. (2013)
	Maternal separation (MS) SD rats	Distilled water	Fluoxetine (5.0 mg/kg)	SNS decoction (2.5 g, 5.0 and 10.0 g/kg/d)	30 days	improve the damage of synapses and mitochondria, reduce the decrease of ATP in hippocampus, and reverse the expression levels of postsynaptic density 95 (PSD-95), synaptophysin (SYN), mitofusin 2 (Mfn2), dynamin-related protein 1 (Drp1), and fission 1 (Fis1) proteins	Deng et al. (2022)
	MS + CUMS SD rats	Distilled water	Fluoxetine (5.0 mg/kg)	SNS decoction (2.5 g, 5.0 and 10.0 g/kg/d)	40 days	upregulate the expression of Calcium sensitive receptor (CaSR), protein kinase C (PKC), and p-ERK1/2 in the HIP and PFC; improve synaptic plasticity by activation of the CaSR-PKC-ERK signaling pathway	Shen et al. (2020)
	MS Wistar rats	Distilled water	Fluoxetine (5.0 g/kg)	SNS decoction (2.5, 5, 10 g/kg/d)	34 days	upregulate the expression of Serotonin 1A (5-HT1A) receptor, p- cAMP response element-binding protein (CREB), and BDNF in the hippocampus, regulate the BDNF/PKA/CREB pathway	Cao et al. (2019)
	reserpine-induced depressive SD rats	Normal saline	Venlafaxine (15 mg/kg)	SNS decoction (0.75, 1.5, and 3.0 g/kg)	14 days	decrease IL-1b, IL-6, and TNF-a expression in the serum, liver, and hippocampus; change the protein levels of NF-kB, BDNF, and TrkB in the hippocampus; alter CYP450 enzymatic activity in the liver	Zong et al. (2019)
	SD rats	Distilled water	Fluoxetine (20.0 g/kg)	SNS decoction (1, 2 and 4 g/kg)	7 days	decreased serum CORT and plasma adrenocorticotropin (ACTH) levels, modulate the hypothalamus-pituitary-adrenal axis, elevate the mRNA expression of hippocampal glucocorticoid receptors	Wei et al. (2016)

**TABLE 2:** Clinical researches of formulas. (XYS: Xiaoyaosan; CSS: Chaihu-Shugan-San; SNS: Si-Ni-San).

Formula	Treatment group	Control group	Samplesize treatment/control	Treatment time	Results	References
XYS	Jiawei Xiaoyao pill (6 g/d, 2 times/d)	Placebo (6 g/d, 2 times/d)	70/71	4 weeks	After treatment: HAMD ↓, HAMA ↓ Treatment group compared to control group: HAMD ↓, HAMA ↓ (the difference was not significant)	Chen et al. (2020c)
	Xiaoyaosan	—	21	6 weeks (21 patients) and 8 weeks (8 patients)	After treatment: HAMD ↓	Tian et al. (2014)
	Jiawei Xiaoyao capsule (10 g* 2/d) + sertraline placebo	sertraline (50 mg/d) + Jiawei Xiaoyao placebo	95/105	8 weeks	After treatment: HAMD ↓, HAMA ↓ Treatment group compared to control group: HAMD ↓, HAMA ↓, Clinical Global Impression efficacy index (CGI-EI) ↑	Su et al. (2019)
	Xiaoyaosan (1 dose/d)	—	17	8 weeks	After treatment: HAMD ↓	Liu et al. (2020b)
	Xiaoyao pill (3 g* 2/d)	Placebo pill (3 g* 2/d)	90/90	8 weeks	After treatment: Hamilton Rating Scale for Depression (HRSD) ↓ Treatment group compared to control group: HRSD ↓	Du et al. (2014)
	Jiawei Xiaoyao granule (10 g*2/d) + Sertraline (50 mg/d)	Sertraline (50 mg/d)	78/62	4 weeks	After treatment: HAMD ↓ Treatment group compared to control group: HAMD ↓	Wu et al. (2019)
	Jiawei Xiaoyaosan (1 dose/d) + Metformin sustained release tablets (0.5 g*2/d) + Deanxit (1 tablet*2/d)	Metformin sustained release tablets (0.5 g*2/d) + Deanxit (1 tablet*2/d)	35/36	8 weeks	After treatment: HAMD ↓, HAMA ↓ Treatment group compared to control group: HAMD ↓, HAMA ↓	Li and Kang (2019)
	Xiaoyao pill (6 g*3/d) + Venlafaxine (75 mg/d, gradually adjust to 150–225 mg/d)	Venlafaxine (75 mg/d, gradually adjust to 150–225 mg/d)	39/37	6 weeks	After treatment: HAMD ↓ Treatment group compared to control group: HAMD ↓	Ma and Li (2019)
	Xiaoyao pill (8 pills*3/d) + Donepezil (5 mg/d)	Donepezil (5 mg/d)	45/45	8 weeks	After treatment: Mini Mental State Examination (MMSE) ↑, activities of daily life ability (ADL) ↑, HAMD ↓, dopamine (DA) ↑, BDNF ↑, Hcy ↓ Treatment group compared to control group: MMSE ↑, ADL ↑, HAMD ↓, DA ↑, BDNF ↑, Hcy ↓	Shen et al. (2018)
	Xiaoyao pill (8 pills*3/d) + Olaxitam injection + Donepezil	Escitalopram Oxalate (10 mg/d) + Olaxitam injection + Donepezil	50/50	8 weeks	After treatment: CSDD ↓, MMES ↑, ADL ↓, BNDF ↑, NE ↑, DA ↑, 5-HT ↑, S100B ↓, Hcy ↓ Treatment group compared to control group: 5-HT ↑, adverse reaction ↓	Shen et al. (2019)
	Xiaoyaosan (150 mL*2/d) + paroxetine (20 mg/d)	Paroxetine (20 mg/d)	32/30	6 weeks	After treatment: Vm_ACA_, Vm_MCA_, Vm_PCA_ (the mean blood velocity of the anterior cerebral artery, the middle cerebral Artery, and the posterior cerebral artery) ↓; Visual analogue scale (VAS) ↓, HeadacheImpact Test-6 (HIT-6) ↓, HAMD-24 ↓ Treatment group compared to control group: Vm_ACA_, Vm_MCA_, Vm_PCA_ ↓; VAS ↓, HIT-6 ↓, HAMD-24 ↓	Zhang et al. (2023)
CSS	citalopram hydrobromide (20 mg/d) + Modified Chaihu Shugan Granule (1 dose/d)	citalopram hydrobromide (20 mg/d)	50/50	8 weeks	After treatment: HAMD ↓; Barthel Index (BI), BDNF ↑ Treatment group compared to control group: HAMD ↓; BI ↑, BDNF ↑	Hu and Sheng (2016)
	Chaihu Shugan Tang (1 dose/d)	Fluoxetine (20 mg/d)	43/43	6 weeks	After treatment: HAMD ↓; DA, 5-HT, NE, Gly ↑; Asp, Glu ↓ Treatment group compared to control group: DA, 5-HT, NE, Gly ↑; Asp, Glu ↓	Shi et al. (2018)
	Lamotrigine + Chaihu Shugan Decoction (1 dose/d)	Lamotrigine (1 dose/d)	24/24	12 weeks	After treatment: HAMD ↓; NA、5-HT ↑ Treatment group compared to control group: HAMD ↓; NA、5-HT ↑	Zhang and Gou (2018)
	Sertraline (25 mg/d, gradually increased to 50–100 mg/d over 2 weeks) + Chaihu Shugan Powder (1 dose/d)	Sertraline (25 mg/d, gradually increased to 50–100 mg/d over 2 weeks)	42/42	8 weeks	After treatment: S100β ↓, BDNF ↑; CRP, IL-6, TNF-α ↓; HAMD, NIHSS, ADL ↓, MMSE ↑ Treatment group compared to control group: S100β ↓, BDNF ↑; CRP, IL-6, TNF-α ↓; HAMD, NIHSS, ADL ↓, MMSE ↑	Jiang et al. (2017b)
	Chaihu Shugan powder (1 dose/d)	Chinese medicine simulated granules (1 dose/d)	63/32	7 weeks	After treatment: SAS、SDS ↓ Treatment group compared to control group: SAS、SDS ↓	Lyu et al. (2022)
SNS	Sini powder (2 times/d; 30 min after breakfast and 30 min before bed)	paroxetine (started at 10 mg/d, increased by 10 mg every 4 days to 40 mg for maintenance)	18/17	4 weeks	After treatment: HAMD‐24 ↓, dim light melatonin onset (DLMO) ↓, phase angle difference (PAD) ↑(SNP group), sleep latency (SL) ↓, sleep efficiency (SE) ↑, total sleep time (TST) ↑(SNP group) Treatment group compared to control group: DLMO ↓, rapid eye movement (REM)↓, non‐REM (NREM) ↑	He et al. (2022)
	Sini powder (1 dose/d)	Duloxetine (20 mg/d)	42/42	15 weeks	After treatment: HAMD ↓, Pittsburgh Sleep Quality Index (PSQI) ↓; IL-1 β ↓, IL-6 ↓, TGF- β 1 ↑ Treatment group compared to control group: HAMD ↓, PSQI ↓; IL-1 β ↓, IL-6 ↓, TGF- β 1 ↑	Guan and Qu (2023)
	Aspirin (100 mg/d), Citicoline Sodium Capsules (200 mg/time, 3 times/d), rosuvastatin (10 mg/d), duloxetine (30 mg/time, 2 times/d) + Modified Sini powder (1 dose/d)	Aspirin (100 mg/d), Citicoline Sodium Capsules (200 mg/time, 3 times/d), rosuvastatin (10 mg/d), duloxetine (30 mg/time, 2 times/d)	47/47	4 weeks	After treatment: Neurological function score (NIHSS) ↓, HAMD ↓; IL-1β ↓, homocysteine (Hcy) ↓, IL-18 ↓; BDNF ↑, basic myelin protein (MBP) ↓ Treatment group compared to control group: NIHSS ↓, HAMD ↓; IL-1β ↓, Hcy ↓, IL-18 ↓; BDNF ↑, MBP ↓	Rao et al. (2021)
	Modified Sini powder (1 dose/d)	Domperidone (12.72 mg/time, 3 times/d), deanxit (1 tablet/time, 2 times/d)	48/48	30 days	After treatment: Motilin(MOT) ↑,gastrin(GAS) ↑, gastric emptying ↑; HAMD ↓ Treatment group compared to control group: MOT ↑,GAS ↑, gastric emptying ↑; HAMD ↓	Xi et al. (2014)

Chaihu Shugan San (CSS), first documented in the Ming Dynasty’s Jingyue Quanshu, is a classical formula in traditional Chinese medicine. As per traditional Chinese medicine theory, it is utilized for the treatment of liver qi stagnation syndrome. This formula synergistically combines *Paeonia lactiflora* Pall. [Paeoniaceae, *Paeonia lactiflora root*] with *Bupleurum chinense* DC. [Apiaceae, *Bupleurum chinense root*], complemented by *Cyperus rotundus* L. [Cyperaceae, *C. rotundus rhizomaa et root*], *Conioselinum anthriscoides ‘Chuanxiong’* [Apiaceae, *C. anthriscoides ‘Chuanxiong’ rhizoma et root*], *Citrus reticulata* Blanco [Rutaceae, *C. reticulata pericarp*], *Citrus × aurantium* L. [Rutaceae, *Citrus aurantium fruits*], and *Glycyrrhiza uralensis* Fisch. ex DC. [Fabaceae, *Glycyrrhiza uralensis radix et rhizoma*] (Gao et al., 2022). Modern research has substantiated its significant efficacy in alleviating depression (Wang et al., 2012; Qiu et al., 2014a; Qiu et al., 2014b) (Table 2), although the underlying mechanisms remain elusive. In recent years, extensive studies have explored the antidepressant mechanisms of CSS (Table 1). Bioinformatics analyses indicate that CSS addresses depression through multiple targets and pathways, including the regulation of 110 differentially expressed proteins (DEPs) in the hippocampus and the modulation of various neurotransmitter transport and circulation (Zhu et al., 2021). Furthermore, CSS has been shown to reverse hyperactivity of the HPA axis, enhance cerebral blood flow perfusion, and mitigate depressive symptoms. Research indicates that it significantly increases regional cerebral blood flow (rCBF) in patients suffering from major depressive disorder (Vangu et al., 2003), and single-photon emission computed tomography (SPECT) has validated its efficacy in improving cerebral perfusion deficits, correlating with clinical symptom relief (Qiu et al., 2014a). Numerous studies have demonstrated that CSS enhances monoamine neurotransmitter levels, regulates BDNF, and modulates the BDNF-TrkB-ERK/Akt signaling pathway, thereby exerting antidepressant effects (Qiu et al., 2014b; Li et al., 2009; Chen X. Q. et al., 2018; Fan et al., 2023). Additionally, CSS shows promise in treating post-stroke depression by downregulating pro-inflammatory factors, including interleukin-1 (IL-1), interleukin-6 (IL-6), and tumor necrosis factor-α (TNF-α), as well as associated proteins such as STAT3 and PTEN, while upregulating glycogen synthase kinase 3β (GSK3β). It also regulates microglial polarization and mitigates neuroinflammation through the activation of the JAK/STAT3-GSK3β/PTEN/Akt pathway (Fan et al., 2023). Recent research indicates that CSS downregulates microRNA-124 (miR-124), upregulates target genes such as mitogen-activated protein kinase 14 (MAPK14) and glutamate ionotropic receptor AMPA subunit 3 (Gria3), promotes synaptic reconstruction in the hippocampus of CUMS rats, and ameliorates depressive behavior (Liu Q. et al., 2018). In conclusion, CSS, with Paeonia lactiflora *Pall.* [Paeoniaceae, Paeonia lactiflora root] as its primary metabolite, exerts antidepressant effects through multiple targets and pathways.

SiniSan(SNS), originating from the Shanghan Lun of the Han Dynasty, consists of equal parts of white *Paeonia lactiflora* Pall. [Paeoniaceae, *Paeonia lactiflora root*], *Bupleurum chinense* DC. [Apiaceae, *Bupleurum chinense root*], *Citrus × aurantium* L. [Rutaceae, *C. aurantium fruits*], and *Glycyrrhiza uralensis* Fisch. ex DC. [Fabaceae, *Glycyrrhiza uralensis radix et rhizoma*], making it a standard formula for treating depression caused by liver qi stagnation (Shen et al., 2023). Recent clinical trials have confirmed its significant efficacy in addressing various types of depression (Zhang Qi et al., 2022) (Table 2). Animal studies indicate that SNS significantly elevates the levels of serotonin (5-HT), norepinephrine (NE), and dopamine (DA) in the brains of depressed mice, while concurrently reducing serum cortisol (CORT) levels (Yi et al., 2013) (Table 1). Prior research has established a link between mitochondrial diseases, depression, and diminished hippocampal synaptic plasticity and neuronal atrophy, all of which are associated with abnormal mitochondrial function and reduced ATP levels in the hippocampus and prefrontal cortex under stress conditions (Aleksandrova et al., 2019; Malberg et al., 2021; Tartt et al., 2022). The SNS has been shown to enhance synaptic plasticity and alleviate mitochondrial damage in maternally separated (MS) female rats under stress, prevent ATP depletion in the hippocampus, increase the postsynaptic density (PSD) at glutamatergic neurotransmitter transmission sites—specifically at asymmetric synapses—and inhibit the overexpression of key proteins involved in mitochondrial fission and fusion (Deng et al., 2022). SNS can activate the CaSR-PKC-ERK signaling pathway, upregulate the expression of serotonin 1α receptors (5-HT1α), phosphorylated cAMP response element-binding protein (p-CREB), and BDNF in the hippocampus, thereby alleviating depressive and anxious behaviors in MS rats (Shen et al., 2020; Cao et al., 2019). Furthermore, SNS enhances the activity of cytochrome P450-related enzymes (CYP1A2, CYP2D1, CYP2E1, CYP3A2), decreases the expression of inflammatory cytokines (IL-1β, IL-6, TNF-α) in reserpine-induced depressive rats, and increases the protein levels of nuclear factor-κB (NF-κB), BDNF, and tyrosine kinase B (TrkB) in the hippocampus. Its mechanism of action in treating depression may be associated with the modulation of CYP450 enzyme activity in the liver (Zong et al., 2019). In models of CORT-induced neuronal damage, SNS regulates the expression of autophagy-related proteins, activates the phosphoinositide 3-kinase/protein kinase B/mammalian target of rapamycin (PI3K/AKT/mTOR) pathway, and prevents excessive autophagy (Zhang M. et al., 2021). Additionally, its extract can modulate the HPA axis, diminish acute stress-induced elevations in serum CORT and plasma corticotropin-releasing hormone (CRH), and reverse the reduction in hippocampal glucocorticoid receptor (GR) mRNA levels (Wei et al., 2016).

Existing evidence indicates that PF, as the primary active metabolite, exerts certain antidepressant effects in formulas such as XYS, CSS, and SNS; however, it accounts for only a portion of the overall effect. The antidepressant efficacy of these traditional Chinese medicine formulas arises from the integrated synergistic effects of multiple plant metabolites, pathways, and targets. While PF could serve as one of the quality markers, it should not be simplistically equated with the evaluation of the entire formulation. Current studies, however, are unable to distinguish between the isolated effects of PF and the overall effects of the formula. We propose that future research adopt a progressive strategy of “formula - combination - metabolite”. Moreover, the research could utilize an integrated approach of network pharmacology, metabolomics, and microbiomics. According to PF as a reference, researchers can systematically compare the differences in targets and molecular mechanisms among single drugs, the combination of PF and core protein, and the entire formula. This approach may clarify the precise target and contribution weight of PF within the formula and further explore the mechanisms of other key active metabolites and their synergistic effects within the formula.

## 7 Research limitations and future prospects

**FIGURE 6:**
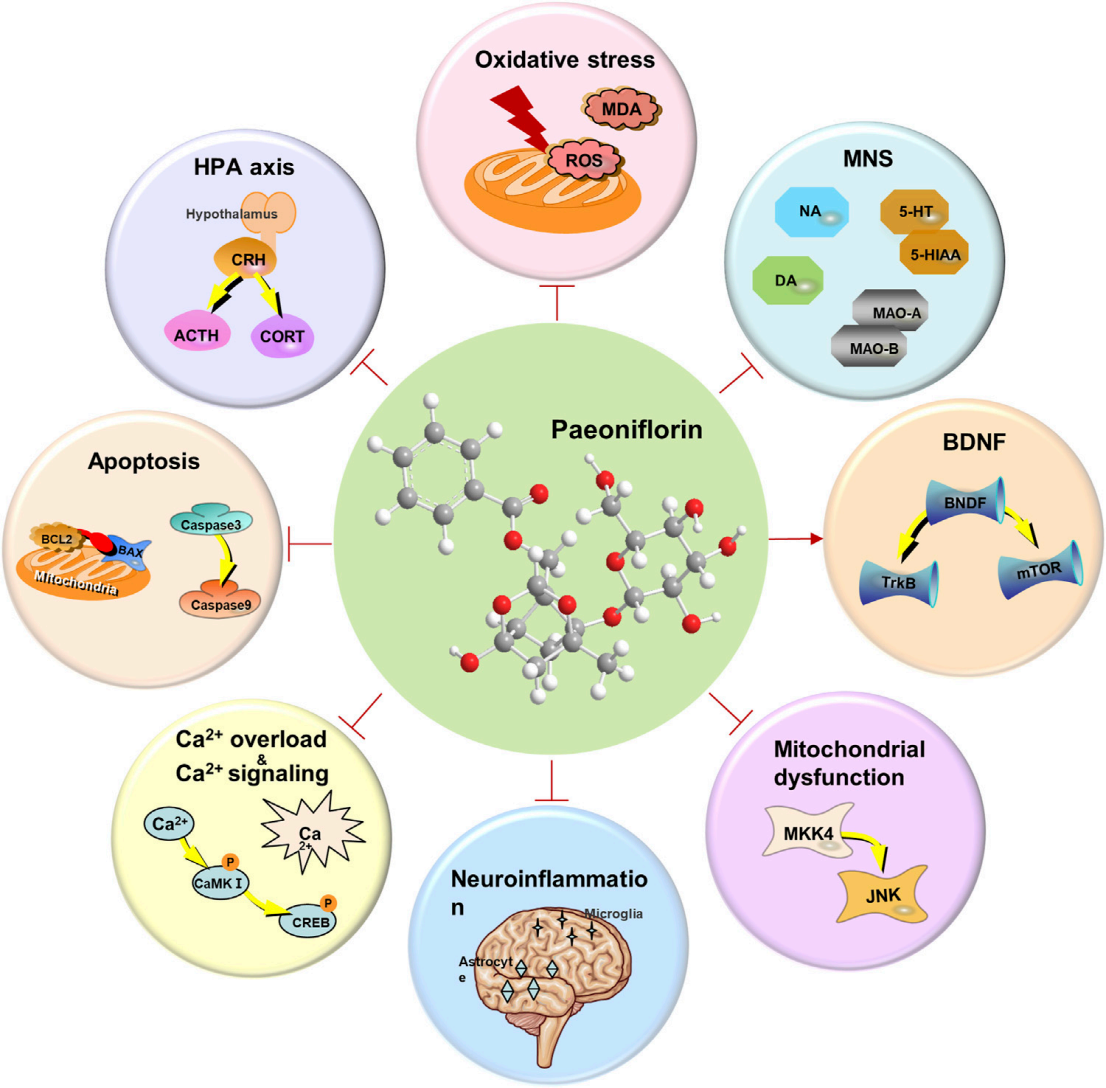
The mechanism of paeoniflorin exerting antidepressant activity.

In recent years, greater emphasis has been placed on mental health and wellbeing. However, depression has become more common, and the incidence has gradually increased. Depression exhibits characteristics that are not confined to a specific age group and may be in the elderly, youth, or even children. Depression, characterized by symptoms such as negative mood, persistent low mood, self-depreciation, and loss of interest in life, seriously affects the life and health of individuals. Although a variety of antidepressant drugs have been developed, including TCAs, MAOIs, and selective 5-HT reuptake inhibitors, they still have defects such as constipation, urinary retention, cardiovascular risk, gastrointestinal symptoms, emotional agitation, or tolerance (Marwaha et al., 2023; Peng et al., 2015). Fortunately, some time-honored formulas documented in traditional Chinese medicine, such as the XYS, have demonstrated promising antidepressant efficacy for millennia (Fathinezhad et al., 2019; Liu L. et al., 2015). Modern studies have found that PF, the main plant metabolite contained in these formulas, may be one of the important factors for good antidepressant and neuroprotective effects (Wu et al., 2024). PF is a bioactive metabolite with the characteristic of being both medicinal and edible, widely present in plant-based foods. It not only possesses nutritional functions but also exerts positive effects on human health through various bioactive mechanisms. In animal experiments, PF has shown good preventive and therapeutic effects on the menopausal depression model, CUMS model, FST, and PPD model. Moreover, a wide range of researchers have focused on exploring the intrinsic molecular mechanisms by which PF exerts antidepressant effects. PF may exert antidepressant activity through a variety of mechanisms, such as preventing the overactivity of the HPA axis, regulating the monoaminergic nervous system, maintaining normal calcium homeostasis and calcium signaling pathway, inhibiting oxidative stress and apoptosis, and regulating the expression of neurotrophic factors in the brain (Figure 6). However, some urgent problems of PF are still to be solved (Guo et al., 2022). Although PF, a water-soluble plant metabolite, could be conveniently administered to patients in the clinic. These qualities, such as the chemical instability, the necessity of storing at low temperatures, avoiding alkaline environments, and the low oral bioavailability, have led to some limitations in application (Yu et al., 2019). The explorations study has found that PF may help patients recover physical and mental health through multi-pathway and multi-target ways. Unfortunately, the mechanism has been extensively studied but not deeply, leading to more potential pharmacological effects and targets that may not be found yet (Hong et al., 2022; Wang X. L. et al., 2021). Particularly, the differences in analytical methods and composition still have a certain impact on the results of most studies with the medicine formula containing PF, which suggests that researchers should consider the importance of developing the standardization of research methods. Besides, the evaluation of side effects and toxicity of PF on target organs is rarely reported. Therefore, long-term systematic drug safety trials are encouraged to achieve the optimal level of safety. Most importantly, clinical trials and data on PF for depression are still lacking. Future studies may require well-designed and adequate clinical trials to explore the deeper intrinsic mechanisms of PF on the one hand and to look forward to designing more specific treatment regimens to achieve optimal efficacy on the other. Indeed, clinical trials with a single natural plant metabolite should also consider the selection of reference reagents and the development of validated indicator judgments. We suggest that in antidepressant clinical trials of PF, study designers should also consider the setting of the Run-in period while focusing on blinding and randomization, which could avoid confounding factors that may interfere with the results of the trial and thus improve the reliability of the study.

## 8 Conclusion

Overall, PF, as a bioactive plant metabolite with dual purposes in medicine and food, not only provides new insights for the treatment of depression but also offers strong support for the health benefits of bioactive metabolites in plant-based foods. In this paper, we present the first narrative review of the mechanisms of PF in antidepressant therapy and the antidepressant applications of traditional compounding. Compared to a systematic review, this study is a better reference for researchers who could contribute to the study of the molecular pathways of PF in depression, as well as the formulation. In particular, these constructive comments on the methodology of PF in antidepressant clinical trials provide a basis for the systematic evaluation of its safety and efficacy in the clinic.
